# Book Reviews

**Published:** 1826-08

**Authors:** 


### CRITICAL ANALYSES.

Quæ laudanda forent, et quæ culpanda, vicissim

Illa, prius, cretâ; mox hæc, carbone, *notamus.*—PERSIUS.

*A Treatise on Diet: with a View to establish, on practical grounds, a System of Rules, for the Prevention and Cure of the Diseases incident to a Disordered State of the Digestive Functions.* By J. A. PARIS, M.D. F.R.S. Fellow of the Royal College of Physicians, &c. &c.—8vo. pp. 307. London: T. and G. Underwood. 1826.

"Some physiologists will have it that the stomach is a mill,—others that it is a fermenting vat, others, again, that it is a stew-pan;—but, in my view of the matter, it is neither a mill, a fermenting vat, nor a stew-pan—but a STOMACH, gentlemen, a STOMACH."—*Manuscript Note from Hunter's Lectures.*

NOTHING can be more certain than that the discrepancy of opinion which so frequently obtains amongst physicians, upon the most trifling subjects connected with their art, tends very effectually to strengthen the scepticism of the public, both with regard to the rationality of their doctrines and the efficacy of their remedies. We meet with a very striking illustration of the uncertainty of medical opinion, in the conflicting doctrines which have been, and are, maintained upon the subject of dietetics. Notwithstanding the labours that have been expended upon the subject, much must yet be effected before we can claim the confidence which is to be gained only by unanimity. Our author, then, approaches a subject which, however frequently considered, is by no means exhausted; and it is but a fair tribute to his acknowledged ability to consider him fully equal to the task he has undertaken. The very motto he has selected relieved us from the apprehension of having to contend with still another hypothesis of the precise manner in which the stomach performs its functions, with fanciful similarities between the process of digestion and some chemical or mechanical operation.

Dr. PARIS does not assert that we possess no works of merit upon the subject of Dietetics: the valuable treatise of Dr. FORDYCE would alone be sufficient to repel the charge. But, since the period in which that and other able works were published, physiology, as well as chemistry, has advanced with rapid strides; "pathology has thrown off the mystic veil with which the humoral doctrine had invested her, and the views, as well as the language of medical science, have undergone corresponding revolutions. Facts alone remain unchanged; but those are so buried in the ruins of the fallen fabric, that, unless they be rescued from the confused mass, their intrinsic value must be entirely lost:

any work, therefore, carefully collated, with the view of accomplishing such an object, even should it present but little novelty, must prove an acceptable offering to the intelligent part of the community."

We agree with Dr. Paris, that every author is conventionally allowed to state the theme of his discussion in his own language; but really the formal introduction of a detailed "Anatomical View of the Digestive Organs," and the "Physiological History of Digestion," which occupy one of the three parts which constitute the volume, was not called for. The suspicion the author himself entertains upon this subject, is doubtless correct: "The relation of a tale which has been so often told may, perhaps, appear to many as not only superfluous, but reprehensible." The custom of the present day, of prefacing the consideration of the diseases or derangements of a particular part by its anatomical and physiological description, would, in our opinion, be "more honoured in the breach than the observance." The professional reader, at least, is not supposed to require such elementary instruction; and the unnecessary repetitions to which the adoption of such a plan must unavoidably give rise, would alone induce us to complain of its adoption. We pass over the first part, then, not that we deny its importance, but because we may fairly presume our readers to be acquainted with the subjects upon which it treats; and that it would be an act of supererogation on our parts to give a lecture upon "the complicated machinery by which nature extracts blood from food."

The second part of the volume is occupied by the discussion of many important subjects connected with the "materia alimentaria." Although, says our author, it is true that those bodies which have possessed life can alone be strictly considered as affording aliment to animals, yet there are many inorganic substances, as water, salt, lime, &c. which, although incapable by themselves of nourishing, appear, when administered in conjunction with the former, to contribute essentially to nutrition. "The consideration, therefore, of the *materia alimentaria* necessarily embraces not only the *substantive* agents above stated, but those which, from their modus operandi, are entitled to the distinctive appellation of alimentary *adjectives*. Under the former division will be arranged all the varieties of animal and vegetable food: under the latter, the class of condiments will merit our attention." (P. 90.)

As every description of food, whether derived from the animal or vegetable kingdom, is converted into blood, it may be inferred that the ultimate effect of all aliments must be virtually the same. The several species differ only from each other in the quantity of nutriment they afford,—in the comparative degree of stimulus they impart to the organs through which they pass,—and in the proportion of vital energy they require for their assimilation.

" Were the degree of excitement which attends the digestion of a meal commensurate with the labour imposed upon the organs which perform it, less irritation and heat would attend the digestion of animal than of vegetable food; for, in the one case, the aliment already possesses a composition analogous to that of the structure which it is designed to supply, and requires little more than division and depuration; whereas, in the other, a complicated series of decompositions and recompositions must be effected before the matter can be animalised, or assimilated to the body. But the *digestive fever*, if we may be allowed the use of that expression, and the complexity of the alimentary changes, would appear, in every case, to bear an inverse relation to each other. This must depend upon the fact of animal food affording a more highly animalised chyle, or a greater proportion of that principle which is essentially nutritive, as well as upon the immediate stimulus which the alimentary nerves receive from its contact. In hot countries, therefore, or during the heats of summer, we are instinctively led to prefer vegetable food; and we accordingly find that the inhabitants of tropical climates select a diet of this description: the Bramins in India, and the people of the Canary Islands, Brazils, &c. live almost entirely on herbage, grains, and roots; while those of the north use little besides animal food. On account of the superior nutritive power of animal matter, it is equally evident that the degree of bodily exertion, or exercise, sustained by an individual, should not be overlooked in an attempt to adjust the proportion in which animal and vegetable food should be mixed. Persons of sedentary habits are oppressed, and ultimately become diseased, from the excess of nutriment which a full diet of animal food will occasion: such a condition, by some process not understood, is best corrected by acescent vegetables. It is well known that artisans and labourers, in the confined manufactories of large towns, suffer prodigiously in their health whenever a failure occurs in the crops of common fruits: this fact was remarkably striking in the years 1804 and 1805. Young children* and growing youths generally thrive upon a generous diet of animal food: the excess of nutritive matter is consumed in the development of the body, and, if properly digested, imparts strength without repletion. Adults and old persons comparatively require but a small proportion of aliment, unless the nutritive they think just the contrary of all this.

* " The aliment of almost every animal, in its first stage of life, is composed of animal matter: even graminivorous birds are nourished by the yolk for several days after being hatched."

movement be accelerated by violent exercise and hard labour." (P. 93.)

The exclusive value of animal food has sometimes been urged, and the utility of its admixture with vegetable matter has been denied; while, to support the proposition, reference has been made to the "rude health and herculean strength of our hardy ancestors." But, although it would appear that the British aborigines were not conversant with the cultivation of the ground, and that they subsisted chiefly on flesh and milk, before any valid conclusion can be drawn from this circumstance, the habits of the people must be compared with those of their descendants.

"We learn from the London Bills, that scurvy raged to such an excess in the seventeenth century, as to have occasioned a very great mortality: at this period the art of gardening had not long been introduced. It appears that the most common articles of the kitchen garden, such as cabbages, were not cultivated in England until the reign of Catharine of Arragon: indeed, we are told that this queen could not procure a salad, until a gardener was sent for from the Netherlands to raise it. Since the change thus happily introduced into our diet, the ravages of the scurvy are unknown.

"It follows, then, that in our climate a diet of animal food cannot, with safety, be exclusively employed. It is too highly stimulant; the springs of life are urged on too fast, and disease necessarily follows. There may, nevertheless, exist certain states of the system which require such a preternatural stimulus; and the physician may therefore confine his patient to an animal regimen, with as much propriety as he would prescribe opium or any other remedy. By a parity of reasoning, the exclusive use of vegetable food may be shown to be inconsistent with the acknowledged principles of dietetics, and to be incapable of conveying a nourishment sufficiently stimulating for the active exertions which belong to our present civilised condition. At the same time it must be allowed, that an adherence to vegetable diet is usually productive of far less evil than that which follows the use of an exclusively animal regimen." (P. 95.)

No sooner had it been demonstrated by the science of chemistry that azote was an essential element of the animal body, and exists in it more abundantly than in plants, than it became a subject of investigation to determine from whence that element was derived: whether from the atmosphere by the process of respiration or absorption, or both; from the action of life itself; or from different articles of food. The experiments which are well known to have been instituted by the indefatigable Magendie, and the conclusion derived from them,—namely, that the azote of the organs is produced by the food, and consequently that no substance which does not contain this principle can support life, are not deemed satisfactory by Dr. Paris, who thus discusses the question:

"Now, giving all due credit to the accuracy and good faith with which these experiments were performed, what do their results show? That the azot of the organs is produced by the food, says M. Magendie, and consequently that no substance which does not contain this principle can support life. By no means: they merely prove that an animal cannot be supported by highly-concentrated aliment. In contradiction to the theory of Magendie, we know that sugar is highly nutritive, provided it be properly mixed with a quantity of substantial viands. It is certain that, in the process of making hay, if well performed, it will be found that the nutritive matter is greatly increased by the partial conversion of the cruder mucilaginous sap into a substance analogous to sugar; as we find that animals thrive faster with this food, and prefer it to that which is left on the ground, and found in a state of self-made hay. Horses fed on concentrated aliment are invariably liable to various diseases, originating from diseased action in the stomach; and hence arise broken wind, as it is termed, staggers, blindness, &c. The intolerable fetid odour of sulphuretted hydrogen gas, perceptible when post-horses are fed with oats and beans only, cannot have escaped observation; and it affords sufficient proof of the mischief which arises from a too-concentrated diet. The same remark applies to men; and I shall have occasion to show hereafter that the use of chocolate, butter, cream, sugar, and rich sauces, without a due admixture of bread, potatoes, and other less nutritive aliments, is invariably attended with disordered digestion. Unless the taste be vitiated by habit, there exists an instinctive aversion to such food.

——— 'The prudent taste
Rejects, like bane, such loathsome lusciousness.'

The Kamtschadales are frequently compelled to live on fish oil, but they judiciously form it into a paste with saw-dust, or the rasped fibres of indigenous plants." (P. 99.)

It is a curious fact, that most of the animals fed by Magendie on *non-azotised* substances, exhibited before death an ulcer in the cornea. The membrane was in some instances destroyed, and the humors of the eye escaped.

There is as much variety in the arrangement of the different articles of food to which we look for support, as in the host of diseases which lead to our destruction; and perhaps all the classifications which have been proposed have been erected upon equally arbitrary foundations. Every author views the subject in a manner peculiar to the notions he has preconceived. The mere arrangement, perhaps, is not a matter of importance; "for, however greatly the roads of our pursuit may vary, we must ultimately arrive at the same goal,"—provided there be any truth in our dietetic researches, or any natural affinity between the objects of our classification. With regard to the nature of these proximate principles of organic matter, upon the presence of which the nutritive qualities depend, Chemistry has satisfactorily demonstrated them to be *fibrin, albumen, gelatin, oil and fat, gluten, fecula, or starch mucilage, sugar, acids, &c.*

The author considers it necessary "to caution the reader against the popular error, of regarding the terms digestible and nutritive as synonymous and convertible. A substance may be highly nutritive, and yet be digested with difficulty: that is to say, it may require all the powers of the digestive organs to convert it into chyle, and yet, when so converted, it may afford a principle of highly-restorative energy: this is the case with some of the fatty and oily aliments. On the contrary, there are substances which apparently pass out of the stomach with sufficient readiness, but afford but little comparative support to the body."

It is very correctly stated, that, if we infer the degree of digestibility of a substance by its known solubility out of the stomach, we shall fall into many errors. Such, however, has been the mode of reasoning adopted by some inquirers into the subject, and hence the fallacious inferences that have been deduced by them. Gosse, of Geneva, for instance, confounds solubility with digestibility, which in itself is sufficient to invalidate his reasoning.

"The healthy stomach disposes most readily and effectually of solid food, of a certain specific degree of density, which may be termed its *digestive texture:* if it exceed this, it will require a greater length of time, and more active powers, to complete its chymification; and, if it approaches too nearly to a gelatinous condition, the stomach will be equally impeded in its operations. It is, perhaps, not possible to appreciate or express the exact degree of firmness which will confer the highest order of digestibility upon food: indeed, this zero may vary in different individuals; but we are taught by experience that no meat is so digestible as tender mutton: when well conditioned, it appears to possess that degree of consistence which is most congenial to the stomach; and in this country it is perhaps more universally used than any other animal food. Wedder mutton, or the flesh of the castrated animal, is in perfection at five years, and is by far the sweetest and most digestible; ewe mutton is best at two years' old. Beef appears to be not so easy of digestion; its texture is firmer, but it is equally nutritive. Much, however, will depend upon the period which has elapsed since the death of the animal, and more upon the method of cookery. In short, it would be worse than useless to attempt the construction of any scale to represent the nutritive and digestive qualities of the different species of food: the observations here introduced are merely noticed for the sake of illustrating those general principles, whose application can alone afford us any rational theory of diet.

"It will not be difficult to understand why a certain texture and coherence of the aliment should confer upon it digestibility, or otherwise. Its conversion into chyme is effected by the solvent power of the gastric juice, aided by the *churning* which it undergoes by the motions of the stomach; and, unless the substance introduced possess a suitable degree of firmness, it will not yield to such motions. This is the case with soups and other liquid aliments: in such cases, therefore, nature removes the watery part before digestion can be carried forward. It is on this account that oils are digested with so much difficulty; and it is probable that jellies and other glutinous matters, although containing the elements of nourishment in the highest state of concentration, are not digested without considerable difficulty: in the first place, on account of their evading the grappling powers of the stomach; and in the next, in consequence of their tenacity opposing the absorption of their more fluid parts. For these reasons I maintain, that the addition of isinglass, and other glutinous matter, to animal broths, with a view to render them more nutritive to invalids, is a pernicious custom." (P. 103.)

It is well known that the texture of food will vary according to the wild or domesticated state of the animal: that of the former is more dense, although highly nutritive. The flesh of the female is more delicate than that of the entire male. It is generally believed that the flavour of the female is improved by removing the ovaries, or *spaying* them. Every day the testes are permitted to remain, even though totally inactive with regard to their proper function, is injurious to the delicacy of the veal of the bull-calf; and an animal which is not castrated until after puberty, always retains much of the coarseness of the entire male. The quality of the meat is also affected by the mode of killing the animal. Hunted animals, and those which die a lingering death, are particularly tender: hence the custom of gradually bleeding calves to death, as practised in this country; of whipping pigs to death, as said to be done by the Germans; of baiting bulls, &c. The action of vinegar given to an animal some hours before killing it, "is also known to be capable of rendering its flesh less tough. It is a common practice in the country to give a spoonful of this acid to poultry, when they are intended for the immediate service of the table." We never heard of this mode of obviating the toughness of recently killed poultry, but shall certainly not fail to recommend its adoption to our country friends, Incipient putrefaction tends most effectually to lessen the rigidity of the animal fibre.

The modifying powers of cookery, however, which our author next proceeds to examine, are infinitely more capable of influencing the texture of our food, and consequently its degree of digestibility, than any of the circumstances just mentioned. "By cookery, alimentary substances undergo a twofold change: their principles are *chemically* modified, and their textures *mechanically* changed. The extent and nature, however, of these changes will greatly depend upon the manner in which heat has been applied to them; and if we inquire into the culinary history of different countries, we shall trace its connexion with the fuel most accessible to them. This fact readily explains the prevalence of the peculiar species of cookery which distinguishes the French table, and which has no reference, as some have imagined, to the dietetic theory, or superior refinement, of the inhabitants." (P. 106.)

We confess we doubt the correctness of this explanation of the peculiar cookery of different countries, and should have been gratified by the mention of the sources from whence the opinion has been derived. The observations of Dr. Paris upon the different modes of preparing food for the table, may not be marked by much novelty, but they are certainly worthy the attention of every practitioner.

"*Boiling*.—By this operation the principles not properly soluble are rendered softer, more pulpy, and consequently easier of digestion; but the meat, at the same time, is deprived of some of its nutritive properties, by the removal of a portion of its soluble constituents: the albumen and gelatin are also acted upon; the former being solidified, and the latter converted into a gelatinous substance. If, therefore, our meat be boiled too long or too fast, we shall obtain, where the albumen predominates, as in beef, a hard and indigestible mass, like an overboiled egg; or, where the gelatin predominates, as in young meats, such as veal, a gelatinous substance, equally injurious to the digestive organs. Young and viscid food, therefore, as veal, chickens, &c. are more wholesome when roasted than when boiled, and are easier digested. Dr. Prout has very justly remarked, that the boiling temperature is too high for a great many of the processes of cooking, and that a lower temperature and a greater time, or a species of infusion, are better adapted for most of them. This is notorious with substances intended to be stewed, which, even in cookery-books, are directed to be boiled slowly, (that is, not at all,) and for a considerable time. The ignorance and prejudice existing on these points is very great, and combated with difficulty; yet, when we take into account their importance, and how intimately they are connected with health, they will be found to deserve no small share of our attention.* The loss occasioned by boiling partly depends upon the melting of the fat, but chiefly from the solution of the gelatine and osmazone: mutton generally loses about one-fifth, and beef about one-fourth, of its original weight. Boiling is particularly applicable to vegetables, rendering them more soluble in the stomach, and depriving them of a considerable quantity of air, so injurious to weak stomachs. But, even in this case, the operation may be carried to an injurious extent: thus, potatoes are frequently boiled to the state of a dry, insipid powder, instead of being preserved in that state in which the parts of which they are composed are rendered soft and gelatinous, so as to retain their shape, yet be very easily separated. On the other hand, the cabbage tribe and carrots are frequently not boiled long enough, in which state they are highly indigestible. In conducting this process, it is necessary to pay some attention to the quality of the water employed: thus, mutton boiled in hard water is more tender and juicy than when soft water is used; while vegetables, on the contrary, are rendered harder and less digestible when boiled in hard water.

" *Roasting.*—By this process the fibrine is corrugated, the albumen coagulated, the fat liquefied, and the water evaporated. As the operation proceeds, thé surface becomes first brown, and then scorched; and the tendinous parts are rendered softer and gluey. Care should always be taken that the meat should not be over-done, nor ought it to be under-dressed; for, although in such a state it may contain more nutriment, yet it will be less digestible, on account of the density of its texture. This fact has been satisfactorily proved by the experiments of Spallanzani; and Mr. Hunter observes, that ' boiled, and roasted, and even putrid meat, is easier of digestion than raw." Animal matter loses more by roasting than by boiling: it has been stated above, that by this latter process mutton loses one-fifth, and beef one-fourth; but by roasting, these meats lose about one-third of their weight. In roasting, the loss arises from the melting out of the fat, and the evaporating of the water; but the nutritious matter remains condensed in the cooked solid; whereas, in boiling, the gelatine is partly abstracted. Roast are, therefore, more nutritive than boiled meats.†

" *Frying.*—This process is, perhaps, the most objectionable of

* " Hence it is that beef-tea and mutton-tea are much more calculated for invalids than the broths of these meats.

† " It has been computed that, from the dissipation of the nutritive juices by boiling, one pound of roasted contains as much nourishment as two of boiled meat.

all the culinary operations. The heat is applied through the medium of boiling oil, or fat, which is rendered empyreumatic, and therefore extremely liable to disagree with the stomach.

" *Broiling.*—By this operation, the sudden browning or hardening of the surface prevents the evaporation of the juices of the meat, which imparts a peculiar tenderness to it. It is the form selected, as the most eligible, by those who seek to invigorate themselves by the art of *training.*

" *Baking.*—The peculiarity of this process depends upon the substance being heated in a confined space, which does not permit the escape of the fumes arising from it : the meat is, therefore, from the retention of its juices, rendered more sapid and tender. But baked meats are not so easily digested, on account of the greater retention of their oils, which are, moreover, in an empyreumatic state. Such dishes accordingly require the stimulus of various condiments, to increase the digestive powers of the stomach." (P. 106.)

(To be continued.)

---

*Practical Observations in Surgery: more particularly as regards the Naval and Military Service. Illustrated by Cases, and various Official Documents.* Second Edition, considerably enlarged. By Alex. Copland Hutchison, late Surgeon to the Royal Naval Hospital at Deal; Member of the Medical and Chirurgical Society of London; Corresponding Member of la Société Medicale d'Emulation of Paris; senior Surgeon to the Westminster General Dispensary; Consulting Surgeon to the Royal Metropolitan Infirmary for the Diseases of Children; Surgeon to his Majesty's Dock-yard at Sheerness, and to his Royal Highness the Duke of Clarence, &c.—8vo. pp. 432. London: T. and G. Underwood. 1826.

This publication belongs to a class of works which we hold in much estimation. It is one of real practical utility, containing the experience of a gentleman actively engaged in the public service, as a naval surgeon, during the late war. It is true that the greater portion of this volume has appeared before, under a different title, or in successive Numbers of this and other Journals; but, as these papers are all united in this edition, and many new observations are added, we do not hesitate to recommend its perusal generally to the profession, but more especially so to those young gentlemen who intend to devote themselves to the medical department either of the naval or military service. It is our intention, in the present article, to draw our readers' attention to those points in the volume which strike us as being most useful, and especially to those which appear in print now for the first time.

The first chapter contains remarks on *Amputation*, occupying about 110 pages, and including all the most important circumstances connected with that operation, either prior to its performance, or in the subsequent treatment of the stump. Part of the chapter is controversial, and turns upon the often disputed point as to the period most proper for amputation in gun-shot wounds. It is not our intention to enter into this discussion: dispute, we will not call it, since there cannot, we conceive, be two opinions as to the propriety of immediate amputation in *almost* every case of this sort. In fact, there is no difference of opinion on the subject; since those who believe that it is necessary to wait until the shock received by the constitution has been recovered, are ready to acknowledge that this shock is neither a constant nor a general occurrence; and we are quite convinced that in this light only was the matter viewed by Mr. Guthrie, the surgeon referred to. Mr. Hutchison is a strenuous advocate for immediate amputation, and he backs his opinion by the reports of the surgeons of the ships employed in the bombardment of Algiers, as well as by the strong evidence of many naval surgeons in previous engagements: and certainly they do confirm that opinion most satisfactorily; at the same time they prove that the *shock* so much talked about, although it does now and then occur, is a very rare event, and, even when it takes place, by no means implies the necessity of a lengthened delay. Stimuli may be necessary to produce a reaction in the system, but when that is accomplished, the sooner the amputation is performed the better: nay, there is some evidence to show that, where this reaction could not be produced, the immediate removal of the shattered limb has been followed by the best effects.

The modes of performing the operation and dressing the stump, together with the after-treatment of the patient, come next; and, if there be not very much of novelty in what our author urges upon these points, at least his doctrines are the best that can be offered, and they cannot be too strongly impressed upon the juniors of the profession.

Speaking of the application of the tourniquet, he objects to the size of the pad commonly employed: that which he uses is not thicker than the finger, and this he lays obliquely over the vessel, to preclude the possibility of its being displaced by any direction that it may be found necessary to give to the limb during the operation. The pad is prepared by taking a few turns with a bandage about a rounded piece of deal, of the thickness of a goose-quill, and about one inch and a half in length. The pad should be stitched, to prevent any embarrassment from its unrolling during the application. A principal objection, says our author, to the placing of a large pad over the artery, is that the surface being so much elevated above the circumference of the thigh, a considerable angular space will be left slightly, or not at all, compressed by the circular band of the tourniquet, however closely the instrument may be screwed.

Respecting the operation itself, he says—

"As soon as the bone is cut through, and before the retractors are removed, I make it an invariable rule, whether there be any occasion to use the bone-nippers or not, to take off the asperities, and scrape, or endeavour somewhat to round, the sharp cut edge of the bone with a strong blunt scalpel, in order to prevent the soft parts from being injured, when brought over the end of the bone in forming the stump; which is very apt to happen by the pressure of the adhesive straps and bandage, in doing up the limb. This precaution will be found to be doubly necessary when the amputation is below the knee, owing to the thinness of the integuments covering the ridge of the tibia. Mr. Hey, of Leeds, in this latter case, recommends the *filing* down the ridge of that bone; and military surgeons have been in the practice, of late years, of sawing off a considerable portion of the upper ridge of the tibia after amputation; but I have found nothing more necessary than what has been described above." (P. 74.)

The following directions in dressing the stump are too practically valuable to be omitted:

"In cases where amputation has been performed on one of the great extremities, I never suffer it to be opened, or the dressings removed, before the fifth, and more generally the sixth day, excepting where much tension and inflammation supervene, or an unusual secretion of pus takes place, and is retained by the too close application of the adhesive straps. In the latter case, the outer dressings are removed, and one or two of the straps snipped with a pair of scissors over the incised line, carefully avoiding the ligatures, to permit a free exit of the contained matter: the stump is then closed up as before; but the following day I make it an invariable rule to remove the whole of the dressings.

"The tension and inflammation resulting from amputation, are best combated by the application of emollient cataplasms to the face of the stump, laid over the adhesive plasters, previously relaxed a little, and renewed every two or three hours; or fomentations may be used. If the inflammation has communicated itself to the integuments of the stump upwards, cold, astringent, and evaporating lotions will generally be found the most suitable remedies." (P. 86.)

"The manner of removing the straps of sticking-plaster, too, is, in my opinion, of much consequence to be attended to, especially in the first few dressings; for, if great care be not taken, the slight and newly-formed adhesions may be torn asunder: if, for instance, the strap be stripped off by holding one end at nearly a right angle with the adhering part, the flap will be raised up with it, and thus a separation of the newly united parts be produced.

"My plan is, to reflect the raised end of the strap close down upon the adhering part, and to bring it gently forward with one hand, whilst the removing part of the strap is followed by two fingers of the other, placed upon the skin, which will effectually prevent the occurrence of such an accident; and, when one end is detached from its adhesions, as far as the line of incision on the face of the stump, in like manner the other end is brought down, and the strap wholly removed.

"When the flaps are brought together, and all the straps applied that are deemed necessary to retain them in apposition, I place upon the under flap a thin compress of lint, enveloped in a piece of simple dressing, with the ointment side outwards, (to admit of the more easy removal of the circular strap:) over this a broad circular strap, snipped at the outer edge, to make it apply more closely, is passed, and carried upwards round the member, leaving the cross angles of the stump projecting beyond it. Two smaller straps of sticking-plaster are also passed obliquely over all, crossing them on the face of stump, that the sides of the flaps may be thus kept in closer contact, and thereby union between the inner surfaces more certainly promoted." (P. 88.)

In illustration of the various positions advocated in this chapter, several cases are subjoined, the last of which is that of an African, on whom amputation of the foot, at the tarso-metatarsal articulation, took place. This case is too long for insertion, but we mention it for two reasons: first, because, whenever such an operation is feasible, it enables the patient afterwards to walk with tolerable facility; and, secondly, on account of the following valuable caution:—"I am, notwithstanding, inclined to think that, however successfully we may perform this important operation in England, we should pause before we recommend its adoption in tropical climates; as, from the unavoidable delay of completing the operation, the number of nerves, tendons, and tendinous expansions, that must necessarily be divided with the knife, and the very considerable pain thereby excited, it would, in such situations, be likely to be followed by tetanus." (P. 107.)

The second chapter treats of *Erysipelatous Inflammation,* a disease very common in the naval service, and for the cure of which, Mr. Hutchison some years ago recommended free incisions to be made upon the inflamed part, through the skin and down to the muscles; avoiding, of course, any large veins or blood-vessels that might be in the way. It will not be necessary to make many remarks on this chapter: the mode of cure advocated by Mr. Hutchison has been before the profession long enough to enable surgeons to form a just estimate of its applicability; and we find an able writer, Mr. Cooper, in the *last* edition of his "Dictionary of Surgery," (not in the *second*, as asserted by our author,) reprehending this practice in no very qualified terms. For our own parts, we conceive that Mr. Hutchison might have altered this chapter advantageously, by explaining more precisely the kind of case in which these incisions are most advisable and productive of real advantage. He says, at page 112, "The epidermis, or rete mucosum, has been supposed by some writers to be the particular seat of erysipelas, whilst others confine its morbid action alone to the cellular substance, and a third class to both these parts. My own observation, however, has afforded me convincing proofs that, in the species of the disease now under consideration, its active and destructive influence will be found more especially directed to the skin, and the reticular or condensed cellular substance forming the aponeurosis of the muscles, &c."

It is in those cases where the aponeuroses of muscles are more especially affected, that we conceive the plan of making free incisions to be attended with the greatest benefit; but we are not prepared to extend the practice as far as our author has done.

With these remarks, we pass on to the third chapter, containing observations on *Simulated or Feigned Diseases.* The greater portion of this chapter has been published in two successive Numbers of this Journal, and therefore we need not detain our readers, nor extend this article, by statements with which they must still be familiar; but we may be permitted strongly to recommend the perusal of this part of the work to all beginners, either in the naval or military service, since it will undoubtedly save them from many difficulties and embarrassments, to which all surgeons in the public service are exposed, from the numerous impositions attempted to be practised upon them. In this instance, our author has indeed turned his experience to very good account, and conferred an essential benefit on his medical brethren.

We pass over the following chapter, on *Hospital Gangrene* and *Common Ulcer,* in order to give a more extended account of Chapter Vth, on *Imperforate Anus.* The subject is very interesting, and the paper itself, although read at one of the meetings of the Medico-Chirurgical Society, is presented to the public for the first time.

After noticing the paucity of information on this subject, contained in the surgical writings of former ages, our author recommends a careful examination of every infant upon its birth, by the accoucheur; and, in the event of imperforate anus being discovered, the surgeon ought not, he observes, to operate until the expiration of from twenty-four to sixty hours, though it may be done afterwards with perfect safety, as well as with a prospect of success. A certain degree of distention of the gut is, in fact, necessary to ensure a favourable result. It is important to ascertain, if possible, the distance of the gut from the surface. If the usual hollow between the nates exists, and the situation of the anus is marked by a depression, our author believes the intestine will be found proportionably near, and vice versâ: he is also inclined to think that, where neither mark nor hollow exists, the sphincter ani is wanting. There is also another mode of coming to a just conclusion on this point, which is by tickling the natural situation of the anus with the point of the finger: the infant will then strain, as if to force out the contents, and a protrusion of the part will be the consequence; unless, indeed, it terminates very high up.

The operation necessary for the removal of this malformation is thus described:

"The infant should be placed upon a table, close to its edge, and having its legs and thighs kept up by an assistant, nearly in the same manner as in the lateral operation for the stone; and, if the child be a female, it may be an advantage to pass a director up the vagina, as recommended by Mr. Mantell, of Dover, which will be a guide to the operator, and lessen the chance of his wounding either the vagina or uterus. The surgeon, sitting on a chair before the patient, or with his right knee upon the floor, should make an incision, with a small double-edged scalpel, nearly an inch and a half in length, in the direction of the raphe, provided the gut intended to be cut into be supposed to be at some distance, and immediately upon the situation of the natural anus; taking care to cut upwards and backwards, towards the hollow of the sacrum, lest the bladder of the male or uterus of the female be injured by the instrument, the fore-finger of the left hand being occasionally introduced into the wound, as a further guide to the direction of the incision; and if, after having cut to the depth of about an inch and a half with the scalpel, which will be as deep as can be done with safety with this instrument, there be no appearance of meconium, we should then lay aside the scalpel, and recommend the introduction of the point of a middle-sized common trocar to the bottom of such incision.

"This instrument should be then pushed gently upwards and backwards, inclining rather to the left of the hollow of the sacrum and natural descent of the rectum, as far as the surgeon thinks it prudent, or until he imagines, from a want of resistance to the force employed, that he has penetrated the gut: at the same time that pressure downwards be made by the hand placed upon the abdomen; when the stilette is to be withdrawn, and the contents of the bowel may, possibly, flow through the canula. If, however, on withdrawing the stilette, the surgeon finds he has been deceived, and that no meconium follows its removal, the latter instrument must be reintroduced through the canula, and the triangular point of the instrument forced further upwards, in the direction before stated, as far as will be consistent with safety. The stilette is now again to be withdrawn, when most probably the meconium will follow it.

"This being effected, and the intestine emptied, I should recommend the canula to be retained in the parts for the first two or three days, securing it in its situation by tapes and a napkin, taking care to direct the nurse, in removing the latter, not to disturb this instrument: for I am strongly inclined to believe that many instances of failure in this operation have arisen from not attending to this circumstance, and not preserving a continuity for the excretions between the punctured gut and the external parts, for a sufficient length of time." (P. 259.)

In the event of the instrument not reaching the intestine, even when the surgeon has penetrated as deep as is consistent with prudence, Mr. Hutchison recommends that the surgeon should suggest to the parents of the infant the possibility of making an incision into the cœcum, or into the sigmoid flexure of the colon. That the surgeon may make such a proposal, and that he perhaps is bound to do so, we will not deny; yet what can be more horrible than the contemplation of life sustained by means of an opening so situated. For our own parts, we think that the child so saved would have little cause to be grateful to the operator.

Pursuing the description of the operation in the usual situation, however, our author says, "after the canula of the trochar has remained in the parts one or two days, I should recommend its removal, by passing through it the largest-sized hollow elastic bougie which the canula will admit, and then to withdraw the canula. After the lapse of some days, the hollow instruments may be laid aside, and a sponge-tent used." But our author thinks, after all, that the common bougie is the best, as it does not produce so much irritation as either sponge-tent or gentian-root. After the first fortnight, it will not be necessary to introduce it to so great a depth. At the end of a month, the instrument may be left off in the night; and, after another month, it may be kept in merely for an hour or two in the day. After the parts are completely cicatrised, the introduction of any instrument might be injurious.

We must now pass over a considerable space, not because the matter is uninteresting, but it has already been before the public, and therefore cannot require either analysis or criticism. We pause, however, at page 387, on *Ununited Fractures*. Mr. Hutchison relates the case of a man who was admitted into Deal Hospital, with a fractured humerus: the accident had occurred sixteen weeks prior to his admission, but no union had taken place, and the arm was, from the contraction of the muscles, four inches shorter than that of the opposite side. An opportunity was here afforded for trying the effect of a seton passed between the ends of the fractured bones, and he thus describes the operation, and its result:

"Foiled in every effort hitherto made to afford relief, a seton, consisting of several portions of silk, was now passed through a kind of cartilaginous substance intervening between the sides of the overlapping extremities of the fractured bone, by means of the needle recommended by Mr. Wardrop, but of somewhat smaller dimensions. This seton was occasionally moved, in the hope of exciting some degree of vascular action in the parts affected. Splints and proper bandages were applied, and the most perfect quiescence, with respect to the limb, sedulously enjoined. The wounds were dressed, and all offensive discharges carefully removed, every two or three days; regulating the intervals of this process according to the state of the discharge and the desire of the patient, but always so managed as not wholly to deprive the arm of the support afforded by the splints, &c.

"After persisting in this mode of treatment for about a month, we found ourselves under the painful necessity of withdrawing the seton altogether, from the supervening of a violent attack of erysipelas over the whole member. As soon as the erysipelatous inflammation had subsided, the two portions of bone were found consolidated to a certain extent, the arm only admitting of slight lateral motion at the fractured part; but none whatever in the opposite direction. Some weeks having elapsed, it was my earnest wish to have introduced the seton again; but no entreaties could prevail on the patient to submit. He was therefore invalided and discharged from the hospital; so much benefited as to enable him afterwards to pursue the occupation of a fisherman, in conjunction with his father, to whom he rendered himself extremely useful." (P. 388.)

Two cases of the *Taliacotian operation*; some remarks upon *removing small Antheromatous Tumors*; and a short Essay on *Necrosis*, conclude the volume.

*Observations on the Efficacy of White Mustard Seed, in Affections of the Liver, Internal Organs, and Nervous System; and on the General Management of Health and Life.* By CHARLES TURNER COOKE, Consulting and Operating Surgeon, at Cheltenham. Third Edition.—8vo. pp. 120. Gloucester, 1826.

WE have frequently been asked our opinion of the efficacy of white mustard seed, and, as many of our readers have probably been questioned about it, we have made Mr. COOKE's pamphlet a text on which to graft the very little which requires to be known on this subject.

If the statements contained in this pamphlet be true, then, indeed, may the physician exclaim, with Othello, that his "occupation's gone!" Let our readers peruse the following quotation, and they will perceive the universality of this new, or rather this newly revived, remedy:

"The white mustard seed is an almost certain remedy for all diseases connected with disordered functions of the stomach, liver, and bowels, and as such has been eminently successful in the following (among other) cases, viz.—in tendency of blood to the head, headache, weakness of the eyes and voice, and hoarseness; in asthma, shortness of breath, wheezing, cough, and other distressing affections of the chest; in indigestion, oppression after eating, heartburn, sickness, wind and spasms, cramp, and other uneasy affections of the stomach; in debility, uneasiness, pain and sense of tenderness and soreness in the interior, and particularly at the pit of the stomach, and in pain in the sides and lower part of the body; in scanty and redundant flow of bile, in obstructions that may lead to scirrhous liver, torpor, and other morbid affections of that organ; in deficient perspiration, gravel, scanty and unhealthy state of the urine, and other disorders of the skin and kidneys; in relaxed and irritable bowels, flatulence, and occasional and habitual costiveness; in severe colds, rheumatism, lumbago, spasms and cramp in the body and limbs, partial and general dropsy, palsy, coldness and numbness of the limbs and feet, loss of appetite, failure of sleep, weakness of nerves, depression of spirits, and general debility of the system. In ague, gout, rheumatic fever, epilepsy, scrofula, scurvy, erysipelas or St. Anthony's fire,—in the dreadfully painful affection called tic douloureux,—and in recovery from the small-pox, typhous and scarlet fevers, and other severe disorders connected with a depraved state of the interior,—it has been taken with very considerable advantage. For the long round worms, and the small white ones also, it is incomparably the best remedy hitherto discovered; inasmuch as, both in children and grown-up persons, it not only destroys those reptiles, but, if persevered in long enough to restore the tone of the stomach and bowels, will prevent their recurrence in future." (P. 11.)

Now here is not only a pretty long list of diseases, but many of the most opposite character and tendency, all yielding to the powers of the white mustard seed; so that, whoever is possessed of a packet of this panacea, is in fact master of the whole materia medica. It is true, indeed, that the above passage is not written by a medical man: it is the preamble to a Mr. Turnor's statement of his own case, and is employed by Mr. Cooke, the *consulting and operating surgeon* at Cheltenham, as a kind of text to his work, and is very properly designated by that gentleman, a *simple* narration. We are further told by Mr. Turnor, that three doses of the mustard seed are to be taken in the day,—one *before* breakfast, the second *after* dinner, and the third either at bedtime or an hour before; but why one dose is proper before and the other after a meal, the deponent saith not. Further on, we find it insisted upon that each dose should contain such a quantity as to produce a complete and healthy evacuation of the bowels every day; an effect to which the patient ought to pay particular attention, and in securing which the whole art of exhibiting mustard seed consists.

It is not our intention to analyse this pamphlet: it is sufficient to say that it contains an account of the digestive organs and function, together with some of the diseases to which they are subject, all evidently addressed to the *occasional* reader; and these are backed by a number of anonymous letters, containing accounts of cures, very much in the style of publications which we meet with daily, in the newspapers, and at the corners of the streets.

With regard to the real utility of the mustard seed as an occasional remedy, we shall present our readers with two passages; one from Dr. Paris's Essay on Diet, the other from Dr. Scudamore's recent work.

"The seeds consist of fecula, mucilage, an acrid volatile oil, on which their virtues depend, and which, on standing, deposits a quantity of sulphur; a bland fixed oil, which considerably obtunds the acrimony of the former constituent, and an ammoniacal salt. The fixed and volatile oils may be obtained by expression, and, if the mixture be submitted to the action of alcohol, the latter will be dissolved, and be thus separated from the former. It has been lately discovered, by some experiments conducted in France, that, if the alcoholic solution be evaporated, a solid and crystallisable substance, distinguished by acid properties, may be obtained; and, as sulphur is said to enter into its composition, it has been termed 'sulpho-sinapic acid.' If the whole seeds be macerated in boiling water, we shall at first obtain an insipid mucilage, which, like that of linseed, resides in the skin; but, if the maceration be long continued, the water will become impregnated with matter yielding the odour of sulphuretted hydrogen,—a sufficient proof that a portion of the volatile oil may be thus extracted; and it is probable that this process may even proceed more rapidly in the digestive canal. In administering them, however, as a remedy, we should be cautious to prevent their accumulation in the bowels. A patient, to whom I lately recommended their use, informed me that his evacuations became extremely offensive; so that it is not improbable that a portion of sulphuretted hydrogen may be disengaged during their passage. Their administration evidently requires caution. If any inflammatory irritation exists, they must prove injurious: where, however, there is a sluggish or deficient secretion of the alimentary juices, I have no doubt respecting their utility." (Dr. PARIS *on Diet.*)

"The use of white mustard is at present very popular, and, like all popular remedies, is employed too indiscriminately. Its medicinal power is not a new discovery. CULLEN, in his Materia Medica, vol. ii. p. 171, observes, "As much of the unbruised seeds as an ordinary table-spoon will contain does not prove heating to the stomach, but stimulates the intestinal canal, and commonly proves laxative." Entirely with a view to determine the nature of this article as a medicine, I made an examination of the seeds in their whole state. In a few days after being digested in cold water, they became much enlarged, and the water had powerfully the smell of sulphuretted hydrogen. Submitted to distillation in a common alembic with water, the portions of liquid which first came over possessed the taste of a weak infusion of malt, quite free from pungency. Digested in alcohol, they did not communicate strongly either smell or taste. Some seeds which had passed the alimentary canal were found to be much swollen, and had lost some of their pungency. It is evident from these results, that the seeds, by treatment with these agents, were acted upon with difficulty in their entire state.

"In the Journal de Chemie Medicale, de Pharmacie, &c. No. x. année 1re, MM. HENRY (fils) and GOROT have given an elaborate report of their chemical examination of the mustard-seed, of which the following is the substance:—The seeds yield by expression a fixed and volatile oil, and the latter may be separated from the former by digestion in alcohol. The alcoholic solution, when evaporated, affords a solid and crystallisable substance, possessing acid properties, to which the discoverers have given the name of sulpho-sinapic acid. Sulphur, it is stated, forms a constituent element of this peculiar acid.

"M. JULIA FONTENELLE, in the same Journal, (No. iii.) informs us that, from his researches, he is led to conclude that the mustard-seed owes all its medicinal powers to the volatile oil; for the extraction of which he recommends that the seeds should be reduced to powder, and distilled with eight or ten parts of water.

"It may be considered, therefore, that the properties of the seeds become sufficiently extracted in the stomach and intestinal canal, to excite the mucous membrane to increased secretion, and also to influence the action of the nerves. They are found principally useful to those invalids who suffer from general deficiency of secretion in the intestinal canal, and from nervous langour. I do not conceive that they are so proper for persons of the inflammatory diathesis, and who become easily heated; and I should rather approve of them as an occasional than a constant remedy, for they are not a certain aperient, and I do not think it desirable to subject the canal constantly to this kind of stimulus. If the seeds accumulate very much, some inconvenience may be occasioned by their augmentation of bulk; and, if they be retained in the intestines, some further inconvenience may result from the disengagement of sulphuretted hydrogen." (Dr. Scudamore *on the Stethoscope, &c.*)

